# The role of trust in HPV vaccine uptake among racial and ethnic minorities in the United States: a narrative review

**DOI:** 10.3934/publichealth.2021027

**Published:** 2021-04-06

**Authors:** Nicole Harrington, Yuku Chen, Alana M O'Reilly, Carolyn Y Fang

**Affiliations:** 1 Immersion Science Program, Fox Chase Cancer Center, Philadelphia, PA, USA; 2 Cancer Prevention & Control Program, Fox Chase Cancer Center, Philadelphia, PA, USA; 3 Molecular Therapeutics Program, Fox Chase Cancer Center, Philadelphia, PA, USA

**Keywords:** human papillomavirus, vaccination, trust, United States, racial/ethnic minorities

## Abstract

Despite the clinically proven benefits of the human papillomavirus (HPV) vaccine in preventing cervical and other HPV-associated cancers, vaccination coverage has been suboptimal among adolescents and young adults in the United States (US), particularly among racial and ethnic minority adolescents. Historical legacies, combined with current racial/ethnic disparities in healthcare, may contribute to suboptimal uptake and completion of the HPV vaccine in part through differing levels of trust in doctors and healthcare institutions. The purpose of this narrative review was to characterize trust and its role in decision making about HPV vaccine uptake among US racial and ethnic minorities. We conducted a literature search using the PubMed database, and our search terms yielded 1176 articles. We reviewed 41 full-text articles for eligibility and included 20 articles in this review. These studies used varied measures of trust or mistrust and assessed trust in not only doctors/healthcare providers, but also other sources including pharmaceutical companies, media, and clergy. Our review findings revealed generally high levels of trust in doctors and healthcare providers, but less so in pharmaceutical companies. Mistrust of either healthcare providers, government agencies or pharmaceutical companies was consistently associated with less favorable attitudes and lower vaccine uptake. The downstream effects of mistrust may occur through selected health beliefs regarding the perceived efficacy and safety of the vaccine. Minority groups were more likely to report trust in family members, religious organizations, and media sources compared to their white counterparts. Decision making about vaccine uptake is a multilayered process that involves comparing the perceived benefits of the vaccine against its perceived risks. Understanding how trusted sources can effectively harness the tools of social and traditional media to increase knowledge and awareness may help combat misinformation about the HPV vaccine and improve engagement with diverse communities.

## 1. Introduction

The primary risk factor for cervical cancer is persistent infection with oncogenic subtypes of human papillomavirus (HPV) [1]. The development of prophylactic vaccines to prevent HPV infection represents a significant milestone in the prevention of cervical cancer. In 2006, the US Food and Drug Administration (FDA) approved the first HPV vaccine for administration to females 9-26 years of age for the prevention of cervical cancer [2],[3]; subsequently in 2011, this recommendation was expanded to include males between 9-26 years of age. However, despite the clinically proven benefits of the HPV vaccine, data from national and state surveys indicate varying degrees of HPV vaccine uptake [4],[5]. Nationally, approximately 71.5% of adolescent females and males aged 13–17 years had received one or more doses of the HPV vaccine in 2019 [6]; however, the percentage of adolescents who had completed the multi-dose HPV vaccine series remains low at approximately 56.8% of females and 51.8% of males. Rates of vaccine uptake and completion also vary across racial/ethnic groups and socioeconomic status [6],[7]. A meta-analysis reported that racial and ethnic minorities have higher rates of HPV vaccine initiation than Whites, but are less likely to follow-through and receive all recommended doses of the vaccine [7],[8]. Notably, this disparity does not appear to be explained by differential access to healthcare [9]. Qualitative data are consistent with this finding, as Black and Latino adolescents and their caregivers did not report structural or system-level access barriers to be a factor in HPV vaccine uptake or completion [10].

Instead, other factors may be contributing to suboptimal rates of vaccine uptake and completion in US racial and ethnic minority groups, such as issues of trust in doctors and healthcare institutions. Historically, minority groups have been misused and exploited by healthcare professionals [11], which may make these groups less trusting of doctors and less motivated to get vaccinated or accept vaccination for their children. In the US, widespread racial and ethnic disparities in healthcare, combined with the legacy of the Tuskegee Syphilis Study, have contributed to differential levels of trust in healthcare providers and institutions [12],[13]. As a result, members of minority groups may rely more heavily on other “trusted” voices when making healthcare decisions. In the context of other preventive health services, such as cancer screening, Blacks were less likely to trust their doctor compared to Whites, but they were more likely to report trust in informal sources, such as relatives, friends, and religious organizations [14]. Because trust is associated with utilization of various life-saving preventive health behaviors, including breast and cervical cancer screening [15] and completion of colorectal cancer screening [16], it is critically important to identify the central sources of trusted information in minority and underserved populations. This will enable future studies to partner with trusted sources to effectively disseminate health information to increase uptake and completion of preventive behaviors, including the HPV vaccine. Thus, we conducted a narrative review to characterize trust in various sources, how trust is assessed, and how it may relate to HPV vaccine acceptance and uptake in minority individuals.

## 2. Materials and methods

This narrative review included an analysis of English-language articles published in peer-reviewed journals. The literature search was conducted using the PubMed database for articles from January 2011–September 2020. A combination of the following search terms was used: “human papillomavirus”, “HPV”, “vaccine”, “vaccination”, “immunization”, “trust”, “mistrust”, “racial”, “ethnic”, “minority”, and “United States.” The search terms yielded 1176 articles. We identified an additional 8 articles from reference lists. The first author reviewed the identified records, removed duplicate articles, and screened the titles and abstracts to identify articles for inclusion in the review. After removing duplicate records, we reviewed the titles and abstracts of 848 articles for potential inclusion in this review. Articles were included if they met all of the following criteria: (a) the study sample was US-based; (b) the data collected included an assessment of trust or mistrust; (c) the study sample included racial or ethnic minority individuals; (d) the study focused on HPV vaccine attitudes, intentions, or behavioral uptake; and (e) full text was available in English.

**Figure 1.**
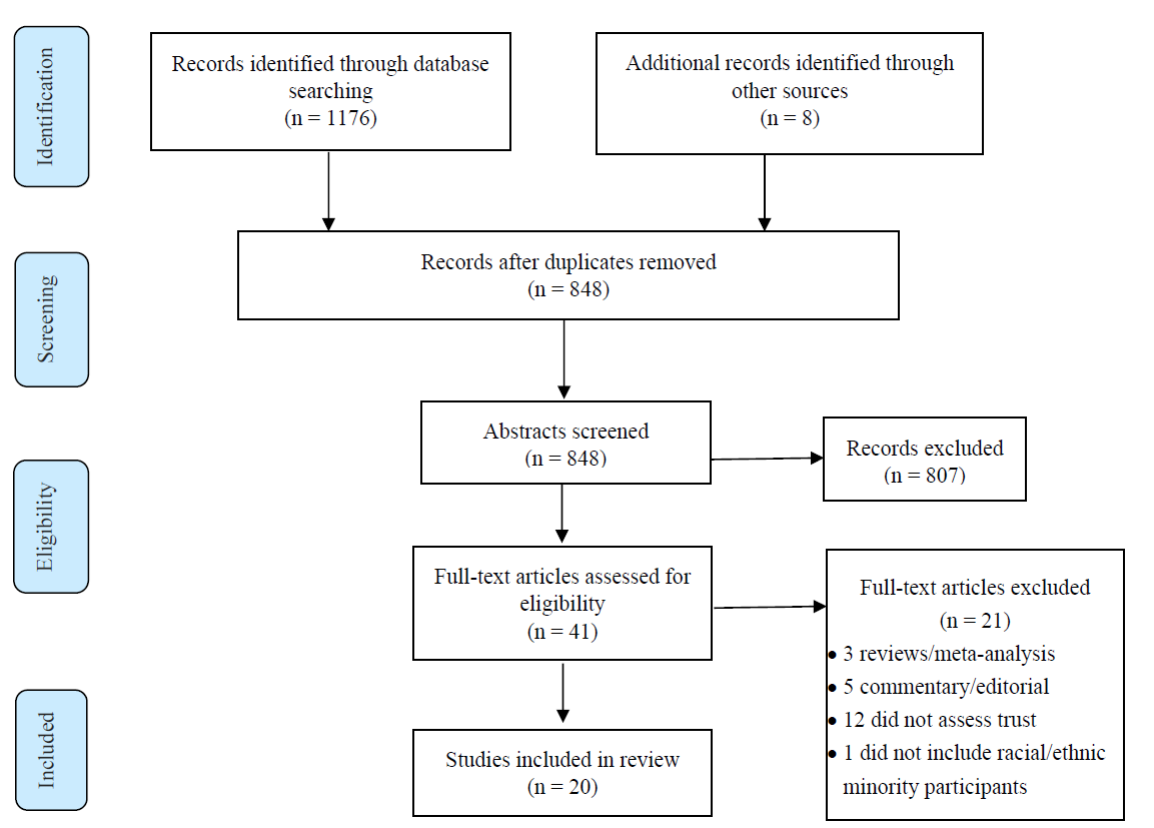
Article selection process.

Of the 848 articles, we excluded 807 because they did not meet the inclusion criteria. We retrieved full-text copies of the remaining 41 articles, which were then reviewed by 3 members of the team for inclusion. Among the 41 articles reviewed, 21 were excluded for the following reasons: meta-analysis or review paper (n = 3); non-empirical paper (i.e. commentary, editorial) (n = 5); did not assess trust (n = 12). One additional article was excluded because the sample did not include any racial/ethnic minority participants (see Figure 1). This screening process resulted in 20 articles that we included in this review.

The first author then reviewed each paper and categorized it according to sample composition and study design. The last author reviewed the categorizations, and any disagreements were discussed among the co-authors until consensus was reached. From each paper, we extracted information about the measures used to assess trust, the sources of trust, the study's primary outcome, and the overall study findings. The co-authors provided critical feedback on the categorization process during multiple meetings held throughout the writing and editing of the manuscript.

## 3. Results

The identified studies assessed trust in various populations, with 5 articles comprised of a general adult population, 11 articles focused on parents or guardians of adolescents (1 of these included a mixture of healthcare providers and key stakeholder organizations along with parents), and 4 articles with a college student sample. With one exception [17], studies were cross-sectional in nature. The majority of studies utilized quantitative surveys, of which five were based on national samples [18]–[22]. Four studies had a qualitative component [23]–[26].

We found that trust was operationalized in a number of ways. Two studies [27],[28] used the 12-item Group-Based Medical Mistrust Scale (GBMMS) [29], a validated and widely used measure of medical mistrust. One paper [30] used the 12-item Parental Trust in Medical Researchers scale [31], which was developed to assess a person's trust in medical researchers. The majority of remaining studies used interview questions (qualitative studies) or single items to assess respondents' levels of trust in various sources including doctors or healthcare professionals. For example, the Health Information National Trends Survey (HINTS) Survey includes an item with the following format: “In general, how much would you trust information about health or medical topics... from a doctor or other health care professional?” Response options ranged from “a lot”, “some”, “a little”, to “not at all”.

Among the 20 articles, 11 (55%) also assessed trust in other sources. Potential other sources included health care systems, pharmaceutical companies, family members, television, professional group websites (such as the American Academy of Pediatrics), government websites (such as the Centers for Disease Control and Prevention [CDC] website), religious organizations, or other media outlets (radio, newspapers, Internet).

Given that different target audiences may have different motivations and concerns pertaining to HPV vaccination, below we present the results grouped by sample composition (see Table 1 for details of each study).

**Table 1.** Summary of studies.

Author (Year)	Sample	Study Design	Trust Measure(s)	Outcome(s)	Trust in Doctor	Trust in other sources	Main Findings
Adult Sample							

Nan et al. (2014) [20]	7674 White, Black, Hispanic adults	Data from 2007 Health Information National Trends Survey (HINTS)	Respondents indicated how much they would trust information about health or medical topics from a doctor or other health care professional, or from government health agencies.	HPV vaccine acceptance for (real or hypothetical) daughter	Yes	Yes	Participants with greater trust in health information provided by a doctor or government health agencies had higher odds of HPV vaccine acceptance for their daughter.
Otanez et al. (2018) [32]	5675 White, Black, Hispanic, and Asian/ Pacific Islander adults	Data from the 2007 Health Information National Trends Survey (HINTS)	Respondents indicated how much they would trust information about health or medical topics from a doctor or other health care professional.	HPV vaccine acceptance for (real or hypothetical) daughter	Yes	No	Almost 70% of respondents reported that they trust information about health or medical topics from a doctor. Doctor trust was a significant predictor of HPV vaccination acceptance, but this association varied by race/ethnicity. Whites and Blacks who distrusted doctors had lower vaccine acceptance than those who trusted doctors. However, among Hispanics, distrust was associated with greater HPV vaccine acceptance.
Ojeaga et al. (2019) [33]	1468 Black and White women	Data from the 2014 Health Information National Trends Survey (HINTS)	How much would you trust information about cancer from: a doctor, government health agencies, family, religious organizations, TV, etc.	Racial/ethnic differences in preferences for source of cancer information	Yes	Yes	Over 95% of all women trusted their doctors for cancer information. Black women were more likely than White women to trust govt health agencies, religious organizations, TV, and family.
Cooper et al. (2017) [18]	1203 White, Black, and Hispanic men	Data from the 2014 Health Information National Trends Survey (HINTS)	How much would you trust information about cancer from a doctor?	Heard of HPV or HPV vaccine	Yes	No	Hispanic men were less likely to trust information about cancer from a doctor than White and Black men. Men who trusted cancer information from a doctor were more likely to have heard of the HPV vaccine.
Teteh et al. (2019) [34]	412 participants from a community forum (86.2% African American)	One-group pretest–posttest study design conducted before and after a series of community forums	How trusting are you of clinical trials? How trusting are you of vaccines? Response options ranged from not trusting at all to very trusting.	Knowledge of HPV and cervical cancer	Yes	No	Perceived knowledge prior to the forums was associated with trust in clinical trials. After the forum, increased perceived knowledge was associated with having trust in vaccines. The positive relationship between perceived knowledge and trust in vaccines observed after the community forums highlights this approach for reducing mistrust.
Parents or Guardians							

Fu et al. (2017) [38]	400 African American parents of adolescents aged 10–12	Parents recruited from waiting rooms of pediatric clinics	Parental trust in sources of vaccine advice, attitudes toward HPV vaccine, and overall impression of the healthcare provider	Receipt of HPV vaccine	Yes	Yes	A greater level of trust in HCPs was associated with higher odds of HPV vaccination. Very strong HCP recommendations were associated with higher odds of vaccination among all subgroups, including those with lower levels of trust.
O'Leary et al. (2018) [56]	244 parents (57.5% Latino/Hispanic) of girls aged 12–15	Mailed survey	Single item “I trust what my health care provider tells me about vaccines”	Non-completion or non-initiation of HPV vaccine	Yes	No	A greater proportion of non-completers agreed or strongly agreed with this statement compared to non-initiators. Spanish-speaking parents were more likely to agree or strongly agree with this statement compared to English-speaking parents.
Cunningham Erves et al. (2017) [30]	256 White and Black parents of adolescent children aged 9–15	Cross-sectional survey	12-item Parental Trust in Medical Researchers	Parental willingness to consent to their child participating in an HPV vaccine clinical trial	Yes	No	Approximately 47% of parents were willing to allow their adolescent to participate in an HPV vaccine trial. Higher scores on the Parental Trust in Medical Researchers scale were positively associated with greater parental willingness.
Cunningham Erves et al. (2018) [37]	237 Black mothers of daughters aged 9-12 (9 mothers also participated in semi-structured interviews)	Sequential mixed methods design	Trusted sources for HPV vaccine information	Mothers' intentions to have daughter vaccinated	Yes	Yes	Many mothers had not received information about the vaccine from their daughter's pediatrician or did not trust the information from them. Mothers did not want information from pharmaceutical companies due to mistrust. Lack of trust in a physician's recommendation was reported as contributing to vaccine hesitancy and low intentions to vaccinate.
Nan et al. (2019) [40]	124 African American parents of children who had not yet been vaccinated for HPV	Survey in community setting	Trust in health information from medical authorities, adapted from the 2007 HINTS	Perceived vaccine efficacy, and attitudes and intentions toward having one's child vaccinated	Yes	Yes	Trust in health information from HCPs was not significantly associated with attitudes or intentions to vaccinate. However, trust in health information from government health agencies was significantly associated with attitudes and intentions. Indirectly, as trust in health information from government agencies decreased, perceived vaccine efficacy decreased, which in turn was associated with decreased attitudes toward HPV vaccination and intentions to have one's child vaccinated.
Painter et al. (2019) [26]	30 uninsured, Latin American immigrant female caregivers of adolescent girls aged 11–17	Semi-structured interviews	Open-ended questions assessed: Whose opinion do you trust on whether your daughter should get vaccines?	Decision making about vaccine uptake	Yes	Yes	The most trusted source of information was a doctor. Mothers described trusting doctors and complying with doctors' suggestions as the major determinants of vaccine uptake for their daughters. Other trusted sources mentioned were spouse/family members, the internet, and media outlets.
Galbraith-Gyan et al. (2019) [25]	African American parents (n = 30) and their adolescent daughters (n = 34)	Qualitative interviews	Open-ended items to assess factors influencing vaccine uptake	HPV vaccine beliefs and acceptance	No	Yes	One parent reported mistrust of political figures advocating for mandating the HPV vaccine.
Clark et al. (2014) [24]	25 Black women who were primary caregivers for girls and young women aged 7 to 20	Focus groups	Trusted sources for HPV information		Yes	Yes	Most trusted source of information was doctors. However, participants also indicated that they commonly double check or verify information obtained from their doctors. The second most trusted source was from family members (mother or older relative). Other trusted sources include the Internet, friends who work in healthcare, and information from community health centers.
Hennebery et al. (2020) [39]	102 guardians of girls ages 12–17 (67% Black); and 149 young adult women ages 18–26 (77% Black)	Surveys were conducted in urban and suburban Obstetrics & Gynecology and Pediatric clinics	Sources of medical information	Initiation of HPV vaccine	Yes	Yes	HCPs were among the most trusted sources of information on the HPV vaccine for 95% of both groups (guardians and young women). However, young women were less likely than guardians to indicate that healthcare providers were the main influences when making decisions about vaccination, and were more likely to report friends, relatives or their spouse as key sources of influence.
Dilley et al. (2018) [36]	7 parents (29% minority including Black, Latinx, and Asian); 19 healthcare providers and other stakeholders (26% minority)	Parents and other stakeholders participated in qualitative interviews	Qualitative interview questions focused on provider HPV vaccination practices and attitudes, and barriers to and opportunities for vaccination at the system, provider and patient levels.	Identifying barriers to and facilitators of HPV vaccination in Alabama	Yes	No	86% of parents and 67% of nurses found doctors to be trustworthy. Parents noted the importance of trust in their decision-making process. Lack of trust in pharmaceutical companies or the government was noted as a barrier to HPV vaccination.
Lazalde et al. (2018) [19]	1209 White, Black, and Hispanic parents of adolescent children aged 11–17	Online survey using a national sample from a survey research company	Parental trust in dentists to administer the HPV vaccine	Parental comfort with dentist administration of the HPV vaccine	Yes	No	23% of parents reported that they would feel comfortable with their child receiving the HPV vaccine from a dentist. Parents were more likely to be comfortable if they had higher trust in their child's primary care provider.
College Students							

MacArthur (2017) [41]	755 college students	Participants completed paper survey	10-item Health Care Provider Trust Scale; 9-item Revised Health Care System Distrust Scale; 8-item scale of trust in pharmaceutical direct-to-consumer advertising	HPV vaccine intentions	Yes	Yes	Trust in one's HCP was directly and positively associated with HPV vaccine intentions. Indirectly, higher levels of trust in one's HCP was associated with greater perceived vaccine efficacy and perceived severity of HPV/HPV-related disease, which in turn were associated with HPV vaccine intentions.
Kolar et al. (2015) [28]	711 college students including Hispanic (n = 329) and Black women (n = 189)	Web-based survey	Medical mistrust was measured using the 12-item Group-Based Medical Mistrust Scale (GBMMS)	Gender and ethnicity preference for HCP with regard to receiving the HPV vaccine	Yes	No	Medical mistrust varied significantly by race with Black women reporting the highest mistrust, followed by Asian women and Hispanic women. White women had the lowest medical mistrust scores. Among unvaccinated women, higher medical mistrust was associated with a preference to receive HPV vaccine recommendations from a HCP of the same race/ethnicity.
Hernandez et al. (2019) [27]	187 unvaccinated Latina college students*	Web-based survey	Medical mistrust was measured using the 12-item Group-Based Medical Mistrust Scale (GBMMS)	Gender and ethnicity preference for HCP with regard to receiving the HPV vaccine	Yes	No	Higher levels of medical mistrust were significantly associated with a preference for receiving HPV vaccine recommendation from a Latinx HCP.
Bynum et al. (2012) [43]	363 Black female college students	Participants completed paper survey	10-item Health Care System Distrust Scale	HPV vaccine acceptance	Yes	Yes	Health care system distrust was not associated with HPV vaccine acceptability.

### 3.1. US Adults

Studies of US adults primarily utilized data from two separate waves of the Health Information National Trends Survey (HINTS) [18],[20],[32],[33], with one exception, which was based on data from a community forum [34]. Among a sample of 5675 self-identified White, Black, Hispanic, and Asian/Pacific Islander adults who participated in the 2007 HINTS [32], nearly 70% of respondents reported that they trust health information provided by a doctor. Respondents were asked to consider if they would vaccinate a (real or hypothetical) adolescent daughter against HPV. Compared to Whites, Hispanics were 30% more willing to vaccinate, whereas Blacks were almost 20% less willing to vaccinate their daughter. A lack of trust was associated with lower willingness to vaccinate across both Whites and Blacks, but with increasing willingness to vaccinate among Hispanics [32]. Overall, among 7674 White, Black, and Hispanic men and women, respondents who reported greater trust in health information provided by a doctor or government health agencies had higher odds of HPV vaccine acceptance for an adolescent daughter [20].

Data from the 2014 HINTS were analyzed separately for men and women [18],[33]. Among 1468 non-Hispanic Black and non-Hispanic White females, trust in cancer information provided by doctors was uniformly high, with over 95% of all women reporting that they trust their doctors [33]. However, Black women were significantly more likely to trust cancer information obtained from family members, television, religious organizations, and government health agencies than White women. Black women also had lower awareness and knowledge about HPV compared to White women. This analysis of women did not examine whether trust was associated with knowledge or behavior pertaining to HPV vaccination, but potential associations were examined among 1203 White, Black and Hispanic men [18]. Differences in trust were observed among men, with a lower proportion of Hispanic men (58.6%) reporting that they trusted cancer information from a doctor compared to Black (74.3%) and White men (71.0%) [18]. About half of the men (49%) had heard of HPV, and 45% were aware of the HPV vaccine. Men who had “some” or “a lot” of trust were more likely to have heard of the HPV vaccine compared to men who did not trust cancer information from a doctor. In summary, national data suggest that a lower proportion of men trust cancer information from their doctors than women do, which in turn was associated with lower awareness of HPV and the HPV vaccine.

A community study of 412 adults (predominantly Black females) assessed participants' levels of trust in clinical trials and in vaccines prior to and following a community forum about HPV and cervical cancer [34]. Following the forum, greater perceived knowledge about HPV and cervical cancer was associated with greater trust in vaccines [34]. It was suggested that increasing knowledge through educational forums in the community may be an effective approach for increasing trust in vaccines.

### 3.2. Parents/Guardians of adolescents

Qualitative studies conducted with parents of adolescents were consistent in noting that doctors are trusted sources of information and advice with respect to the HPV vaccine [26],[35],[36]. Most parents reported that their doctors are trustworthy and serve as a key influence in the decision-making process about HPV vaccination [26],[36]. In contrast, parents did not trust pharmaceutical companies and perceived that pharmaceutical marketing information impacts doctors' advice to parents [35]. A mixed-methods study of Black mothers of daughters directly linked trust to HPV vaccine intentions, revealing that mothers with low intentions to vaccinate did not trust their child's doctor and did not desire to receive information from pharmaceutical companies due to mistrust [37].

Quantitative survey studies confirmed the qualitative findings. Among parents and guardians, doctors were the most trusted source of information across multiple studies [24],[38]–[40]. Even though doctors were the most preferred source of information on HPV, parents reported that they commonly received information about HPV and the HPV vaccine from television [24]. Other trusted sources that were frequently cited include family members (e.g., mother or older relative), governmental agency websites (e.g., Centers for Disease Control and Prevention [CDC]), friends who work in healthcare, and information from community health centers.

Whether trust is associated with HPV vaccine uptake, however, was mixed. A greater proportion of vaccine acceptors trusted their child's doctor “a lot” compared to vaccine refusers; and more vaccine refusers had “no trust” in governmental agency websites [38]. Interestingly, half of vaccine acceptors did not disagree with the statement that “African Americans are being targeted for HPV vaccine while it is still somewhat experimental”; this proportion was even higher among vaccine refusers (66.3%) [38]. In contrast, despite high levels of parental trust in healthcare providers, two studies reported that trust was not significantly associated with initiation of the HPV vaccine [39] or with attitudes or intentions to vaccinate one's child against HPV [40]. Lower levels of trust in information from government health agencies were associated with more negative attitudes toward HPV vaccination and lower intentions to have one's child vaccinated [40], as expected. This association was mediated by perceived vaccine efficacy, such that lower trust in government health agencies was associated with lower perceived vaccine efficacy, which in turn was associated with less favorable attitudes and lower intentions to have one's child vaccinated.

One study measured parental willingness to allow their child to participate in an HPV vaccine clinical trial [30]. There were no racial differences between Black and White parents in their intentions to have their child vaccinated against HPV, but overall intentions were relatively low with 36.7% of Black parents and 40.4% of White parents reporting that they planned to do so. Further, only 30.7% of African American parents and 48.3% of Caucasian parents stated they would be willing to allow their child to participate in a HPV vaccine clinical trial. Trust in medical researchers, as measured by a 12-item Parental Trust in Medical Researchers scale [30], was associated with greater willingness to allow their child to participate in an HPV vaccine clinical trial. As parental trust in medical researchers increased, willingness to allow their adolescent offspring to participate in an HPV vaccine clinical trial increased [30].

### 3.3. College students

Two studies assessed trust among female college students [27],[28] using the Group-Based Medical Mistrust Scale [29]. Analysis of the entire sample of 711 students revealed that Black women reported significantly higher levels of medical mistrust than Hispanic and White women [28]. There was a trend for women who had not received the HPV vaccine to report higher levels of mistrust compared to those who had been vaccinated. A more targeted examination of unvaccinated women revealed that those with higher mistrust scores also preferred a provider of the same race/ethnicity and gender [28]. Subgroup analyses of unvaccinated Latina college students only (n = 187) confirmed that higher levels of medical mistrust were significantly associated with a preference for a Latino healthcare provider [27].

Similar to the patterns observed among parents, college students reported higher levels of trust in a healthcare provider than in health care systems or pharmaceutical direct-to-consumer advertising (DTCA) [41]. Further, trust in one's healthcare provider was directly and significantly associated with greater intentions to be vaccinated against HPV in the upcoming year. Greater trust was also related to college students' health beliefs about the perceived efficacy of the vaccine and perceived severity of HPV and HPV-related diseases. However, in contrast to these studies, health care system distrust measured using the 10-item Health Care System Distrust scale [42] was not significantly associated with HPV vaccine acceptability among Black female students attending historically black colleges/universities [43].

## 4. Discussion

This narrative review of US-based studies revealed generally high levels of trust in doctors and healthcare providers, but less so in pharmaceutical companies. Trust in one's doctor was frequently, but not always, associated with greater intentions and actual HPV vaccine uptake. However, mistrust of either healthcare providers, government agencies or pharmaceutical companies was consistently associated with less favorable attitudes and lower vaccine uptake.

The downstream effects of mistrust may occur through selected health beliefs regarding the perceived efficacy and safety of the vaccine [44]. Decision making about vaccine uptake and completion is a multilayered process that involves comparing the perceived benefits of the vaccine against its perceived risks. Among vaccine refusers, reasons commonly included not only lack of trust in the healthcare provider, but also inadequate or inaccurate knowledge about the pros and cons of the vaccine [45]. Indeed, misinformation spread on social media and the internet is a concern as these messages can directly impact HPV vaccine decisions [46],[47]. Understanding how trusted sources can effectively harness the tools of social and traditional media to increase knowledge and awareness may help combat misinformation about the HPV vaccine and improve engagement with diverse communities.

Racial differences in trust were explored in only a few studies, and findings differed according to how trust was conceptualized and measured. In national surveys that used items asking respondents how much they trusted information from a doctor, levels of trust were relatively high across racial and ethnic groups with a few exceptions. Hispanic men reported lower levels of trust in information from their doctor compared to White and Black men, and this difference appears to be stable across multiple waves of the HINTS [48]. Black women reported higher levels of trust in other sources such as government health agencies, television, religious organizations, and family members compared to White women, and this finding also remained consistent over time [48]. In contrast, studies that used the validated Group-Based Medical Mistrust Scale (GBMMS) found that Black women had the highest levels of mistrust and White women had the lowest levels [28], and levels of mistrust were higher among unvaccinated women compared to vaccinated women. Although a causal inference cannot be drawn from these cross-sectional studies, prior research has demonstrated that greater medical mistrust is associated with lower adherence to other recommended cancer prevention and screening behaviors such as colorectal cancer screening [49].

The findings from this narrative review highlight several opportunities for health promotion efforts to enhance vaccine uptake and completion rates. First, developing partnerships with community-based organizations or religious organizations to deliver accurate and reliable health information can help increase trust in minority communities, as well as diminish the impact of misinformation that may be presented by other sources. Studies have demonstrated that community forums that provide accurate, linguistically- and culturally-appropriate information to its participants offer a potential approach for enhancing trust [34],[50]. Second, increased education and sensitivity training in medical mistrust for healthcare providers and medical researchers may help facilitate a constructive dialogue about how to acknowledge mistrust, recognize implicit and explicit biases, and begin to foster or deepen trust across relationships with patients and community members [51],[52]. Addressing this challenge head on will be an integral action for transforming the culture and atmosphere of mistrust in medical institutions that has been reported by racial and ethnic minorities.

This review identified consistent patterns of mistrust in relation to HPV vaccine attitudes and intentions, but several limitations warrant mention. First, due to the focus on US racial and ethnic minority populations, studies that did not explicitly contain racial or ethnic minority participants were excluded from the review. However, given the relatively small number of such studies and the observation that the nature of the association between mistrust and HPV vaccine uptake is similar in these samples [35], this decision likely did not alter the pattern of findings reported. Second, the majority of studies included Black participants; although some studies focused on Latinx populations, relatively few studies included Asian Americans, despite national statistics demonstrating that Asians aged 9 to 26 have the lowest levels of HPV vaccine initiation among all racial/ethnic groups [53],[54]. Thus, whether mistrust plays any role in HPV vaccine uptake and completion among Asian Americans remains undefined and is a topic that merits future attention. Finally, most studies were cross-sectional in design and examined vaccine intentions/acceptability or attitudes toward the vaccine as the outcome of interest. Although attitudes and intentions do not always translate directly into actual behavior, longitudinal studies have reported that more favorable attitudes are positively associated with HPV vaccine intentions, which in turn predicted vaccine uptake assessed 10 months later [55]. In addition, qualitative data that reflected on how trust or mistrust factored in parents' decision-making processes also inform our understanding of the temporal nature of these findings, in the absence of longitudinal studies.

## 5. Conclusions

In order to decide whether to undergo HPV vaccination for themselves or their children, people need reliable and accurate information about the vaccine. Whether that information comes from a trusted source can affect individuals' attitudes and intentions to be vaccinated. Although most individuals trust their healthcare provider, other sources that may provide less reliable information are also commonly noted as trusted sources. In addition, more global perceptions of medical mistrust can undermine health promotion efforts. A growing body of literature has explored medical mistrust among racial and ethnic minorities, in particular among US Blacks, and its role in contributing to delays in healthcare utilization and preventive care. Findings from this review demonstrate that an increase in trust correlates with increased knowledge and awareness of the vaccine. Future research designed to evaluate best practices for addressing medical mistrust is needed to address community concerns and to enable healthcare professionals to provide better care for all of their patients.
